# Continuous Invariant-Based Maps of the Cambridge Structural Database

**DOI:** 10.1021/acs.cgd.4c00410

**Published:** 2024-06-20

**Authors:** Daniel E. Widdowson, Vitaliy A. Kurlin

**Affiliations:** Materials Innovation Factory and Department of Computer Science, University of Liverpool, Liverpool L69 3BX, U.K.

## Abstract

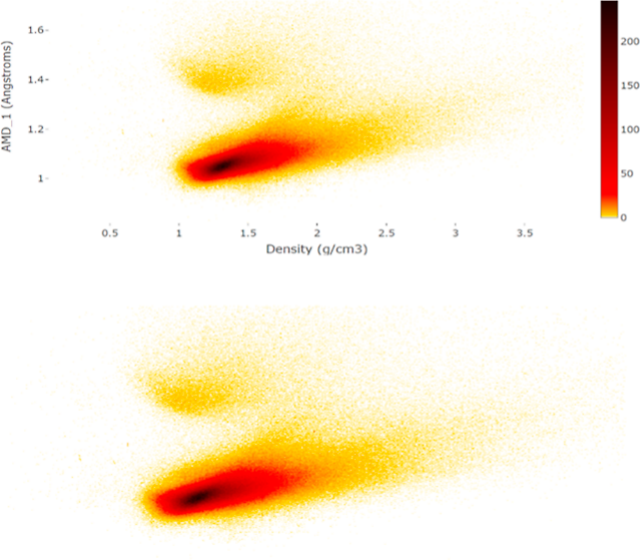

The Cambridge Structural Database (CSD) played a key role in the recently established crystal isometry principle (CRISP). The CRISP says that any real periodic crystal is uniquely determined as a rigid structure by the geometry of its atomic centers without atomic types. Ignoring atomic types allows us to study all periodic crystals in a common space whose continuous nature is justified by the continuity of real-valued coordinates of atoms. Our previous work introduced structural descriptors pointwise distance distributions (PDD) that are invariant under isometry defined as a composition of translations, rotations, and reflections. The PDD invariants distinguished all nonduplicate periodic crystals in the CSD. This paper presents the first continuous maps of the CSD and its important subsets in invariant coordinates that have analytic formulas and physical interpretations. Any existing periodic crystal has a uniquely defined location on these geographic-style maps. Any newly discovered periodic crystals will appear on the same maps without disturbing the past materials.

## Abstract 2

For the Cambridge Structural Database (CSD), the paper describes the first projections of the crystal isometry space of all periodic structures defined as equivalence classes under rigid motion. The coordinates in the resulting maps are invariant descriptors (based on interatomic distances and measured in Angstroms), which continuously change under atomic displacements and distinguish all real periodic crystals in the CSD.

## Introduction: Strong Motivations for Continuous Maps

Crystallography traditionally classified periodic crystals almost exclusively in a discrete way by symmetries. This was a natural approach in the past when only a few crystal structures were known. The classification of 230 space groups was a great achievement in the 19th century by Fedorov^1^ and Schonflies^2^ in 1891. Due to the important work of Olga Kennard, who established the Cambridge Structural Database (CSD) in 1965, and her numerous successors,^3^ the CSD contains now 1.25+ million known materials including more than 830 thousand periodic crystals with no disorder and full atomic geometry. Many more thousands of crystal structures are computationally predicted even for a fixed chemical composition.^4^ These big numbers motivate a finer (stronger) classification into infinitely many classes.

For a simple comparison, triangles can be classified by symmetries as equilateral, isosceles, and generic (nonisosceles). However, geometry did not stop at these three classes and moved on to a much stronger classification, which is now called the side–side–side theorem. This SSS theorem from school geometry says that two triangles can be rigidly matched if and only if they have the same triple of side lengths *a*, *b*, and *c* considered up to all permutations.

**Figure 1:**
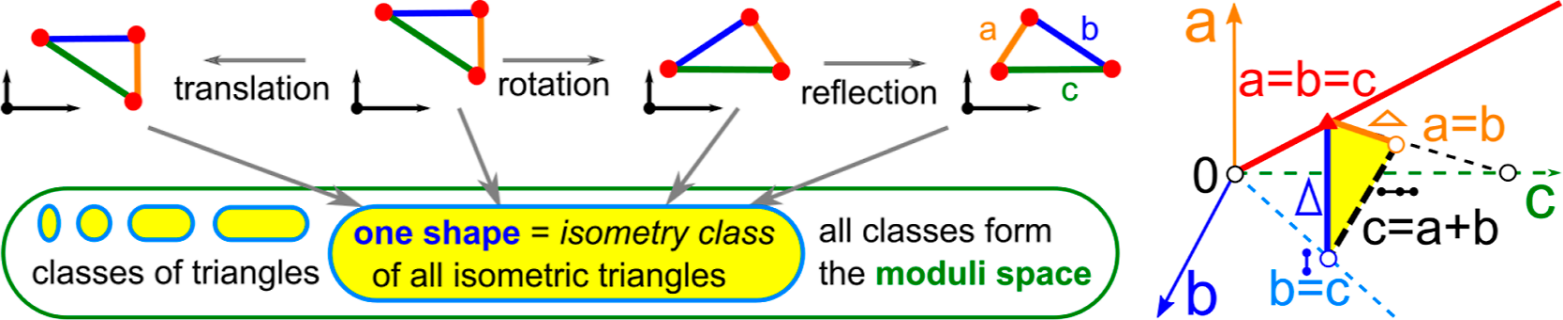
Left: one isometry class consists of all triangles isometric to each other. All such classes form the moduli space of shapes. Right: the moduli space of triangles under isometry is the cone  parametrized by 3 interpoint distances *a*, *b*, and *c*.

In Euclidean space , this rigid matching of triangles is called a congruence and can be obtained as a restriction of isometry, which is any distance-preserving transformation of . Any Euclidean isometry is a composition of translations, rotations, and reflections, as seen in Figure 1. If we exclude reflections, any composition *f* of translations and rotations is called a rigid motion because *f* can be included in a continuous family (motion) , *t* ∈ [0, 1], of isometries such that *f*_1_ = *f* is the given map and *f*_0_ is the identity map.

All rigid shapes of triangles form one infinitely continuous moduli space of classes modulo isometry. The SSS theorem can be rephrased so that the moduli space of triangles is continuously parametrized by an ordered triple of interpoint distances *a*, *b*, and *c* satisfying 0 < *a* ≤ *b* ≤ *c* ≤ *a* + *b*, where the last inequality guarantees the existence of a triangle, e.g. (*a*, *b*, *c*) = (3, 4, 5) uniquely determines a right-angled triangle with side lengths 3, 4, and 5.

Figure 1 visualizes this moduli space as a triangular cone in  with the coordinate axes *a*, *b*, and *c*. The red diagonal {*a* = *b* = *c*} represents all equilateral triangles. Any point in the yellow section determines a unique triangle under isometry and uniform scaling. The dashed line in the plane *c* = *a* + *b* represents degenerate triangles of 3 points in a straight line.

The moduli space of triangles under rigid motion and uniform scaling is the union of two yellow triangles glued along their common boundaries, where triangles are mirror-symmetric, which gives a topological sphere *S*^2^. This continuous classification of triangles under rigid motion (or slightly weaker isometry, possibly with uniform scaling) is much finer (stronger) than the discrete classification by symmetries with only three classes. The classes of all equilateral and isosceles triangles are low-dimensional subspaces of dimensions 1 (diagonal line) and 2 (union of two boundary sides) in the infinite 3-dimensional cone, respectively.

Because crystal structures are solid (or rigid) materials, their most natural equivalence is rigid motion. Indeed, there is little sense in distinguishing crystals related by rigid motion, at least under the same ambient conditions, such as temperature and pressure. Hence, the crucial question “same or different”^5^ has the initial answer same (rigidly equivalent) if they are related by rigid motion. The more practical questions are “how to distinguish all different crystals” and “how to continuously quantify their difference”. As shown in Figure 1, such answers for triangles were known already to Euclid in 300 BC. For instance, the distance between any triangles uniquely represented by triples (*a*, *b*, *c*) and (*a*′, *b*′, *c*′) can be quantified in many continuous ways, the simplest being the Euclidean metric .

Can we rigorously answer the fundamental questions “same or different?” and “if different, how much different?” at least for periodic crystals? The 21st century witnessed an explosive growth of structural databases whose integrity maintenance^6^ now requires continuous tools that can quickly detect numerous near-duplicates as motivated below.

## Discontinuity Challenge of Traditional Crystallography

This section explains the discontinuity of traditional Definition 1 as in Section 8.1.4 of ITA.^7^

### Definition 1 (Unit Cell, Lattice, Motif, Periodic Point Set, Periodic Crystal)

**Figure 2:**
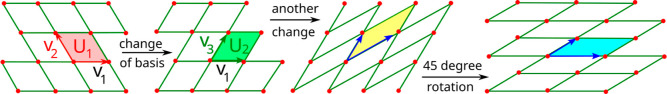
Ambiguity of choosing a basis of a lattice  as introduced in Definition 1.

Any ordered basis of vectors  defines the unit cell (parallelepiped)  and the lattice , as seen in Figure 2. A motif *M* ⊂ *U* is any finite set of points in *U*. A periodic point set S = *M* + Λ is the infinite set of points *p* + *v* for all *p* ∈ *M* and *v* ∈ Λ. In , if each point of *M* is an atom or ion with a chemical element and charge, then the periodic point set *S* with these atomic attributes is called a periodic crystal. ▲

Any lattice can be generated by infinitely many bases. If (*v*_1_, *v*_2_) is one basis of a lattice , then (*Av*_1_, *Av*_2_) is another basis of Λ for any 2 × 2 matrix *A* with integer elements and determinant 1. This ambiguity can be theoretically resolved by a reduced cell.^8^ In dimensions 2 and 3, such a reduced cell can have two types: with all angles between basis vectors acute or with all angles obtuse (nonacute). The hexagonal lattice Λ in Figure 2 has the obtuse and acute cells *U*_1_ and *U*_2_, respectively. While we can choose one of them by allowing the angle 60° and forbidding 120°, all such choices create a discontinuity under almost any perturbation, which was reported^9^ in 1965 and resolved for two-dimensional lattices^10^ in 2022.

When a motif *M* is added to a lattice Λ, the ambiguity of this crystal representation *S* = *M* + Λ significantly increases. First, one can shift a motif within a fixed unit cell *U*, which changes all fractional coordinates of atoms in a basis of *U*, but moves the underlying periodic crystal only by a fixed vector. For highly symmetric crystals, this ambiguity is often resolved by fixing atoms at Wyckoff positions but not for generic crystals with the space group P1. Second, any periodic point set *S* = *M* + Λ can be obtained from an extended motif *M*′ in a scaled-up cell (defining a sublattice Λ′ ⊂ Λ) so that *S* = *M*′ + Λ′. In theory, an extended motif and cell can be scaled down to a primitive cell that has a minimum volume. In practice, a tiny atomic displacement can make an extended cell primitive.

So a Crystallographic Information File (CIF) describing a crystal *S* via a unit cell and motif as in Definition 1 can be considered a single “photograph” of S. The standard settings carefully developed in the International Tables for Crystallography can be informally compared with a standardized passport photo, which sufficed in the 20th century.

In 2024, massive data produced by cheap artificial tools^11^ should be validated by rigorous methods,^12^ such as biometric passports for humans and DNA-style codes for crystals.

A potential attempt to ignore perturbations up to a small threshold ε > 0 by calling any ε-close crystals equivalent (pseudosymmetric) practically shifts^13^ the discontinuity from 0 to ε and theoretically leads to a trivial classification because any crystals can be connected through sufficiently many ε-perturbations. All challenges above for experimental crystals become much worse in simulations because near-duplicates can be easier hidden within big data. Any iterative optimization stops at a close approximation to a local minimum, hence many slightly different approximations can accumulate around the same local mininum.

## Key Concepts for Identifying Near-Duplicate Structures

A rigorous way to answer the question “same or different” is to define “same” (equivalent) crystals so that all their physical or chemical properties are equal. We consider only ideal periodic crystals under the standard ambient conditions such as room temperature and pressure. Because crystal structures are determined in a rigid form, their strongest equivalence in practice is a rigid motion, which is a composition of translations and rotations. Indeed, different rigid structures (even different rigid conformations of the same molecule such as a protein) can have different chemical properties and hence are important to distinguish.

Definition 2 was also proposed in the paper^12^ discussing past ambiguities and was motivated by Carolyn Brock’s call^14^ for “a real-space definition mentioning periodicity”. We propose to separate the concepts of a periodic crystal (an object fixed in space and represented by a CIF) and a crystal structure (a class of all rigidly equivalent crystals). The word crystal refers to a three-dimensional object with chemical attributes, while periodic point sets and their periodic structures (equivalence classes) are based on indistinguishable points in any .

### Definition 2 (Periodic Structure and Crystal Structure Are Classes under Rigid Motion)

In , a periodic structure is a class of all periodic point sets that can be exactly matched to each other by rigid motions of . In , a crystal structure is a class of all periodic crystals that can be exactly matched to each other (with all atomic attributes) by rigid motions. ▲

The earlier attempt^15^ to formalize an equivalence proposed that “crystals are said to be isostructural if they have the same structure but not necessarily the same cell dimensions nor the same chemical composition, and with a “comparable” variability in the atomic coordinates to that of the cell dimensions and chemical composition. For instance, calcite CaCO_3_, sodium nitrate NaNO_3_, and iron borate FeBO_3_ are isostructural. While the IUCr online dictionary does not have the entry “structure”, the definitions of a crystal structure^16^ (in ) and a crystal pattern^17^ (in any ) essentially coincide with a periodic point set and a periodic crystal in Definition 1, respectively. Then, any isostructural crystals should coincide as periodic sets of atoms with fixed positions in  without applying any rigid motion. Even if we interpret the crystal structure in the sense of Definition 2, Table 1 summarizes how many versions of the above compounds differ by their cell lengths and are not equivalent under rigid motion.

**Table 1:** Examples of “Isostructural” Crystals as Defined in the IUCr Online Dictionary^a^

crystal	database references	#	cell lengths *a* = *b*	cell length c	cell angles
CaCO_3_	CSD: FUTWOI...	29	4.945 ≤ *a* ≤ 4.992	16.86 ≤ *c* ≤ 17.81	90, 90, 120°
NaNO_3_	ICSD: 14185...	10	5.071 ≤ *a* ≤ 5.107	16.82 ≤ *c* ≤ 16.83	90, 90, 120°
FeBO_3_	ICSD: 34474...	12	4.621 ≤ *a* ≤ 4.627	14.47 ≤ *c* ≤ 14.5	90, 90, 120°

a Even for a fixed composition, cell parameters of CaCO_3_ vary up to 0.95 Å (from *c* ≈ 16.86 Å in FUTWOI01 to *c* ≈ 17.81 Å in FUTWOI18) among 29 entries in the CSD. All entries in the ICSD are taken for the same space group (R-3cH), room temperature, and standard pressure.

In Pattern Recognition,^18^ the pattern means a class under some equivalence. The structure also deserves a deeper meaning as in Definition 2 because any rotation of a real crystal changes its CIF (coordinate representation) but preserves the underlying crystal structure.

So crystals are considered same (having the same rigid structure) if they can be exactly matched by rigid motion in . The slightly weaker equivalence is isometry, which is any distance-preserving transformation. In , any Euclidean isometry is a rigid motion or its composition with any mirror reflection. If we do not distinguish mirror images, any nonmirror-symmetric crystal defines a larger class under isometry than under rigid motion.

If crystals *S* and *Q* are isometric (matched by an isometry *f* of ), they are rigidly equivalent (matched by rigid motion) or *S* is rigidly equivalent to the mirror image of *Q*. One can separate these cases by checking if *f* preserves the sign of the *n* × *n* determinant consisting of basis vector *v*_1_, ..., *v*_*n*_ in . So it almost suffices to classify crystals under isometry.

To distinguish nonisometric crystals that are not related under isometry, we need an invariant. This concept makes sense for any equivalence, though we consider only isometry.

### Definition 3 (Invariant and Complete Invariant)

An isometry invariant I is a function on periodic point sets from Definition 1 such that if any *S* and *Q* are isometric (denoted by *S* ≃ *Q*), then *I*(*S*) = *I*(*Q*). An isometry invariant is called complete (or injective or separating) if the converse holds for any periodic point sets, i.e., if *I*(*S*) = *I*(*Q*), then *S* ≃ *Q*. ▲

The center of mass of a motif is not invariant because shifting a motif within a unit cell moves the center of mass, so isometric crystals can have different values of any noninvariant. Only an invariant descriptor can distinguish crystals under isometry because Definition 3 implies that if *I*(*S*) ≠ *I*(*Q*), then *S* ≠ *Q*. The motif size (number of points in a primitive cell) and the primitive cell volume are isometry invariants but they are incomplete. Indeed, all lattices have motifs of one point and many of them have the same volumes of primitive cells despite being nonisometric. A complete invariant can be considered a DNA-style code or a materials genome that uniquely identifies any periodic crystal under isometry in .

**Figure 3:**
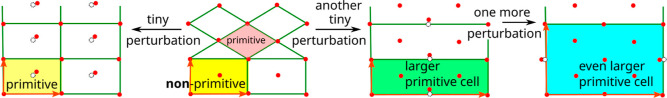
Any primitive or reduced cell arbitrarily extends under almost any perturbation.

Standard (or conventional) settings^19^ for crystal representations were designed to be such a complete invariant, which worked well in the 20th century while structural databases were relatively small. In 2024, near-duplicates can be computer-generated in huge numbers and all represented with very different cells and space groups despite being almost identical. The perturbations of the hexagonal lattice in Figure 3 can be similarly applied to any periodic crystal. Indeed, we can arbitrarily extend a given cell of any crystal and slightly shift a single atom within the initial cell, which makes the extended cell primitive. This discontinuity exists even without extensions for only lattices,^10^ though examples become more complicated.

A rigorous way to quantify the closeness between near-duplicates is to use a continuous metric, which is a distance function between invariant values of crystals, as defined below.

### Definition 4 (Distance Metric)

A metric on value of an invariant *I* of periodic point sets (under isometry) is a function *d* satisfying the following axioms:

(a) coincidence: *d*(*I*(*S*), *I*(*Q*)) = 0 if and only if *I*(*S*) = *I*(*Q*);
(b) symmetry: *d*(*I*(*S*), *I*(*Q*)) = *d*(*I*(*Q*), *I*(*S*)) for any periodic point sets ;
(c) triangle inequality: *d*(*I*(*S*), *I*(*Q*)) + *d*(*I*(*Q*), *I*(*T*)) ≥ *d*(*I*(*S*), *I*(*T*)) for any *S*, *Q*, *T*. ▲

The first coincidence axiom in Definition 4 guarantees that *d* = 0 if and only if *I*(*S*) = *I*(*Q*). Without this axiom, even the zero function *d* = 0 satisfies all other axioms. If the triangle inequality is allowed to fail with any positive error, one can design a distance *d* such that outputs of the *k*-means and DBSCAN algorithms are predetermined^20^ and hence are not trustworthy. Therefore, any clustering should use a distance *d* satisfying all metric axioms.

For the invariant *I*(*S*) equal to the primitive cell volume of *S*, the simplest metric is the absolute difference |*I*(*S*) – (*Q*)|. One can similarly define a distance metric for the complete invariant *I* consisting of a conventional representation *C*(*S*) including atoms at Wyckoff positions in a reduced cell. All these cell-based metrics are discontinuous under almost any perturbation and hence fail to detect the closeness of infinitely many near-duplicates.

A conventional representation *C*(*S*) can be used to detect an isometry *S* ≃ *Q* and define a discrete metric such as  but any such metric is discontinuous. Detecting near-duplicates needs a stronger concept of continuity below.

### Definition 5 (Lipschitz Continuity)

An invariant *I* of periodic point sets is called Lipschitz continuous in a metric *d* if there is a constant λ > 0 such that if any periodic point set  is obtained from *S* by perturbing every point of *S* up to any fixed bound ε ≥ 0 in the Euclidean distance, then the invariants of *S* and *Q* are close in the sense that *d*(*I*(*S*), *I*(*Q*)) ≤ *λε*. ▲

Definitions 3, 4, and 5 help state the continuous classification problem in crystallography.

### Problem 6 (Continuous Isometry Classification)

Find a complete, continuous and quickly computable isometry invariant *I* of all periodic point sets in . In detail, we need

(a) completeness: any periodic point sets are isometric (*S* ≃ *Q*) if and only if *I*(*S*) = *I*(*Q*);
(b) continuity: *I* has a Lipschitz continuous metric *d* in the sense of Definition 5;
(c) reconstruction: any  can be reconstructed (uniquely under isometry) from *I*(*S*);
(d) computability: for a fixed dimension *n*, the invariant *I*, the metric *d*, and a reconstruction of any  from *I*(*S*) can be obtained in polynomial time of the motif size of *S*. ▲

The equality *I*(*S*) = *I*(*Q*) between complete invariants is best checked as *d*(*I*(*S*), *I*(*Q*)) = 0 due to the coincidence axiom in Definition 4. The reconstruction in condition (c) is stronger than the completeness in (a) because the invariant *I* might be too complicated and unsuitable for inverse design of crystals. Conditions (a,b,c) in Problem 6 can be easily satisfied by the abstract invariant *I*(*S*) = {all *Q* isometric to *S*}. The computability in condition (d) makes Problem 6 practically meaningful because the invariant *I* can be used as geographic-style coordinates on the space of isometry classes of all periodic crystals similar to *a*, *b*, and *c* parametrizing the space of triangles in Figure 1. Problem 6 is even harder for rigid motion.

## Progress in Continuous Classifications of Crystals

This section reviews the recent progress in solving Problem 6 for periodic point sets under isometry, which can be replaced with any types of objects under any equivalence. One simple extension is to allow compositions of rigid motion or isometry with uniform scaling in .

The early statement of Problem 6 and a partial solution appeared for lattices^21^ in 2020. Now, Problem 6 is considered a crystallographic example of the general meta-problem in the new area of Geometric Data Science. The ultimate goal is to continuously parametrize the spaces of equivalence classes of data objects on a geographic-style map to visualize structure–property relations and enable an inverse design of new objects by rational exploration.

If our objects are finite sets of unordered points (say, atoms of a molecule) under isometry, Figure 1 illustrates the full solution for *m* = 3 points but the problem for *m* > 3 was solved relatively recently: for nonsingular sets^22^ in 2004 and completely^23^ in 2023. An analogy is the human genome and DNA, whose structure is known^24^ and is considered complete in practice, at least for identifying humans in court trials, though identical twins exist. However, a living organism cannot be easily reconstructed from its DNA yet. So an efficient reconstruction of a periodic set in Problem 6(c) is more challenging than completeness.

For all lattices , Problem 6 was solved^10^ in 2022 (also under rigid motion) by the complete root invariant RI(Λ) with two slight deviations. First, the extra realizability condition explicitly described what values of RI(Λ) can be realized by lattices. Second, if the bound ε on point perturbations is smaller than the minimum quarter-distance between lattice points, one can prove^25^ that the perturbed lattice is a translate of the original one, which easily implies condition (b) in Problem 6. If we perturb not points as in Problem 6(b) but coordinates of basis vectors of Λ up to ε, the root invariant RI(Λ) changes up to  in the Euclidean metric, where *l* is the maximum length of basis vectors. The stronger Lipschitz continuity (with ) seems unrealistic because the rectangular lattices with the ε-close bases (*l*, 0), (0, ε), and (*l*, 0), (0, 2ε) can substantially differ even by unit cell areas *l*ε and 2*l*ε whose difference *l*ε can be arbitrarily large if *l* has no upper bound. For all lattices , conditions (a,c) in Problem 6 hold for the more complicated invariant^26^ whose continuity is being finalized, based on five Voronoi types^27^ instead of 14 Bravais classes.^28^

For general periodic point sets from Definition 1, a strong continuous invariant (the density fingerprint^25^) was obtained by extending the point density to *k*-fold intersections of balls of a variable radius centered at given points. This density fingerprint was proved to be complete for nonsingular periodic point sets (in a general position achieved by almost any perturbation) and Lipschitz continuous in , also computable in polynomial time^29^ in dimensions 2 and 3, but its underlying metric so far has only an approximate algorithm. Later the density fingerprint was shown to be incomplete^30,31^ even in dimension 1 for singular periodic sequences, which were distinguished by the invariants in Definition 7 below.

Definition 7 describes much simpler invariants whose slight modifications will be used as geographic-style coordinates on continuous maps of the CSD in the next section.

### Definition 7 (Distance-Based Invariants PDD)

Let *M* ⊂ *U* be a motif of *m* points in a (not necessarily primitive) unit cell of any periodic point set . Fix an integer *k* ≥ 1.

**Figure 4:**
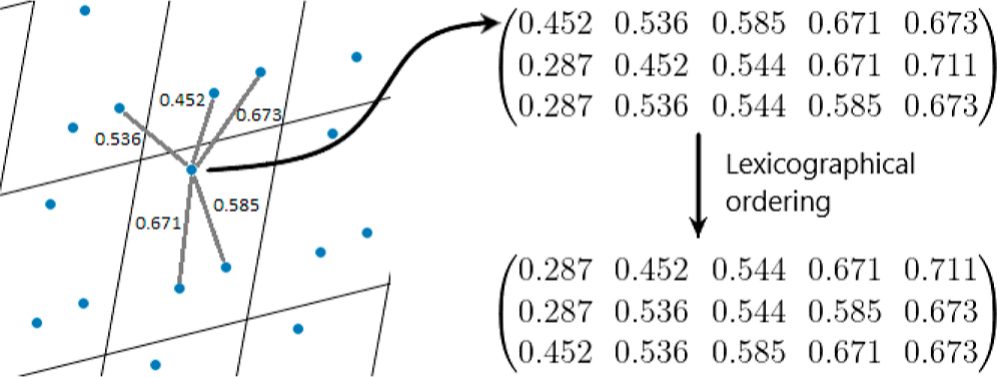
Distances to *k* nearest neighbors in a periodic point set. The lexicographic order is for convenience. The PDD matrices in Definition 7 are compared with unordered rows.

(a) For each point *p* ∈ *M*, the *m* × *k* matrix *D*(*S*; *k*) has one row of *k* distances *d*_1_(*p*) ≤ ··· ≤ *d*_*k*_(*p*) to the *k* nearest neighbors of *p* in the full set *S* not restricted to any cell or a ball of a cutoff radius. If any *l* > 1 rows of *D*(*S*; *k*) coincide, collapse them into a single row and assign the weight *l*/*m*. The resulting matrix with the extra first column of weights is called the PDD^32^ (*S*; *k*). The average minimum distance AMD_*k*_(*S*) is the weighted average of the distances to the *k*-th neighbors, as seen in Figure 4.
(b) Let *V*_*n*_ be the volume of the unit ball in . The point packing coefficient  is the average volume per point, measured in original units such as angstroms.
(c) The average deviation from asymptotic  has original units. The normalized deviation from asymptotic  is unitless. ▲

Figure 4 illustrates the PDD computation and highlights the fact that all *k* neighbors are not restricted to a finite subset whose change may disrupt the output. For any *k* ≥ 1, AMD_*k*_(*S*) is the weighted average of the (*k* + 1)-st column of PDD(*S*; *k*) with the weights from the extra first column, e.g., AMD_1_(*S*) is the average distance to the first neighbor.

Increasing *k* only adds more columns of distances to PDD without changing the previous distances. Hence, *k* is considered not a usual parameter that can substantially affect the result but as a degree of approximation similar to the number of decimal places on a calculator.

The Point Packing Coefficient  measures (the *n*-th root of) the unit cell volume per point normalized by the unit ball volume *V*_*n*_. Roughly speaking, PPC(*S*) inversely proportional to the density of points. Theorem 13 in the AMD paper^33^ proved that the curve of values AMD_*k*_(*S*) approaches  as *k* → + ∞. Hence, the limit behavior of AMD_*k*_(*S*) is largely determined by density and there is no need to substantially increase *k* because the most descriptive information is contained in smaller atomic environments. This asymptotic motivated the modified invariant ADA_*k*_(*S*) in new Definition 7(c). If a periodic point set  is uniformly scaled by a factor *u* > 0, all interpoint distances and hence AMD_*k*_(*S*) and PPC(*S*) are multiplied by *u*, which leaves NDA_*k*_(*S*) invariant.

If we collect all distances from PDD(*S*; *k*) into a single distribution, we get a raw version of the pair distribution function (PDF) as a discrete set of all (infinitely many) interatomic distances. This discrete set can discontinuously change under perturbation when two equal distances become slightly different. In the past, this discontinuity was addressed by taking a convolution with a Gaussian kernel, which converts the discrete PDF into a smooth function. The AMD and PDD invariants resolve this discontinuity at the level of discrete invariants by using Earth Mover’s distance (EMD) without an extra Gaussian deviation parameter.

Writing all distances per point in the PDD makes PDD(*S*; *k*) stronger than PDF, see Example 3.3 in the PDD paper.^32^ The parameter-dependent smoothing of the raw PDF was introduced to guarantee the continuity under perturbations as in Figure 3 when equal distances to neighbors become distinct. For automated comparisons, the smoothed PDF is often uniformly sampled, which creates the counterintuitive pipeline: a discrete set *S* → smoothed PDF → discretely sampled PDF. To avoid this unnecessary smoothing, PDD matrices can be continuously compared in a parameter-free way.

Consider PDD (*S*; *k*) a discrete probability distribution of unordered rows (vectors in ) with weights whose sum is 1. The simplest metric on such distributions is the Earth Mover's Distance (EMD), which came from transportation theory^34^ and has been already applied to comparing chemical compositions,^35^ see Definition 4.1 in the PDD paper.^32^ Briefly, the EMD optimally transforms the rows of one PDD matrix into the rows of another PDD.

**Figure 5:**
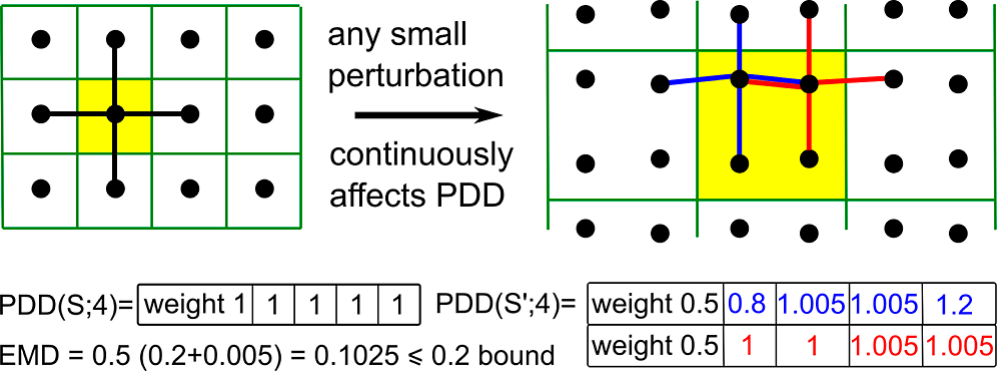
Under a small perturbation by ε = 0.1, the unit cell quadruples but all interpoint distances change by at most 2ε = 0.2, which is averaged by EMD to a value under 2ε.

Figure 5 illustrates the EMD computation when we perturb the unit square lattice *S* to *S*′ by moving both points in every other pair vertically by 0.1 away from each other. As a result, all interpoint distances remain in the range [0.8,1.2]. The only reasonable way to transform the 1-row PDD (*S*; 4) into the 2-row PDD (*S*′; 4) is to split the single row of PDD (*S*; 4) into two-halves, which are compared with the two rows of PDD (*S*′; 4) by (say) the *L*_∞_ metric measuring the maximum absolute deviation of corresponding coordinates. Then, EMD takes the weighted average of the two *L*_∞_ metrics from the row comparisons. Theorem 4.3 in the PDD paper^32^ proved the Lipschitz continuity of PDD (*S*; *k*) in EMD with constant λ = 2.

More complicated atom-centered descriptors involving angles and higher order atom interactions use a cutoff radius and an order of points for angles that may not guarantee the invariance under permutations or completeness when near-duplicates can coincide on a large bounded domain as in Figure 3. Figure 5 explains how the discontinuity is resolved by using only interpoint distances. If a point *p* has two or more neighbors at the same distance then, after a small perturbation, a cutoff ball can include any of them, which can discontinuously affect a finite cluster of points but the *k* smallest distances always change continuously.

The approach through bounded clusters led to the isoset invariant,^36^ which was proved to be complete for all periodic point sets including singular ones in any Euclidean space . The Lipschitz continuous metric on isosets is approximated with a proved error factor.^37^

The strongest theoretical result is the generic completeness and reconstructability for PDD (*S*; *k*) combined with a lattice of *S*. Theorem 4.4 in the PDD paper^32^ proved that any periodic point set  in a general position (outside a singular subspace of measure 0) can be reconstructed uniquely under isometry from a lattice of *S*, which can be given by complete invariants^10,26^ in dimensions *n* ≤ 3, and PDD (*S*; *k*), where *k* should be large enough to include all distances up to 2*R*(*S*). Here, the covering radius *R*(*S*) is the minimum radius of balls that are centered at all points of *S* and cover the full ambient space . Theorem 5.1 in the PDD paper^32^ proved that, for a fixed dimension *n*, the computational time of PDD (*S*; *k*) depends only near-linearly on the motif size *m* and the number *k* of neighbors. The AMD and PDD invariants improved material property predictions^38−40^ on some data sets.

The latest implementation of PDD (*S*; 100) can compare all (more than 830 thousand) periodic crystals from the CSD (with no disorder and full geometric data) through more than 345 billion comparisons in under 1 h on a modest desktop. This ultrafast speed allows us to visualize the CSD in the invariant coordinates on a laptop in real time.

## Crystal Isometry Principle Inspired by Richard Feynman

Definition 2 of a crystal structure as an equivalence class under rigid motion or (slightly weaker) isometry implies that all crystal structures can be studied within a common continuous space of periodic structures. Indeed, ignoring all atomic attributes maps any crystal structure to a periodic structure consisting of only zero-sized points at all atomic centers. Any slightly nonisometric crystals, as listed in Table 1, are represented by close points (isometry classes) in the space of all isometry classes of periodic point sets, which is now called the crystal isometry space . All periodic sets with at most *m* points in a unit cell form a 3*m*-dimensional subspace . Here, 3*m* is the number of fractional coordinates of *m* points, while 6 parameters of a unit cell are counterbalanced by 6-parameter isometries in .

Is it possible that we lose some information when ignoring atomic attributes? The first temptation is to keep at least all chemical elements. Traditional chemistry explored this path for centuries by separately studying organic vs nonorganic compounds and smaller subclasses (intermetallic, semiconductors, and perovskites) to a level of a single composition.

However, fixing chemical elements breaks the continuous space  into many thousands of isolated pieces, one for each composition. These disjointed pieces can contain very different structures, such as diamond and graphite, which does not help distinguish polymorphs that have the same composition but nonequivalent crystal structures.

The AMD^33^ and PDD^32^ papers reported several pairs of (near-) duplicates where all numerical parameters in the CIFs were equal with almost all digits but one atom was replaced with a different one. For example, the CSD entries HIFCAB^41^ and JEPLIA^42^ essentially differ only by replacing Cd with Mn at the same position without any other changes in the unit cell parameters or fractional coordinates. The integrity office at the Cambridge Crystallographic Data Centre (CCDC) checked that their structure factors were also identical and agreed that the found (near-) duplicates need a redetermination with better precision.

The all-vs-all comparisons of only periodic structures (without chemical attributes) for the CSD by PDD (*S*; 100) implied that if real periodic crystals are not isometric, then their periodic structures are not isometric. So ignoring atomic attributes loses no data, i.e., the map {real crystal structures} → {periodic structures} is injective modulo isometry for the CSD. This conclusion confirms our physical intuition that replacing one atom with a different one should perturb distances to neighbors at least slightly. All-vs-all comparisons are being finalized for other experimental databases, such as COD,^43^ ICSD,^44^ and MP.^45^

The resulting crystal isometry principle (CRISP) says that there should be no theoretical obstacle to reconstructing atomic attributes such as chemical elements from a periodic set of only atomic centers if their coordinates are determined with a high enough precision.

**Figure 6:**
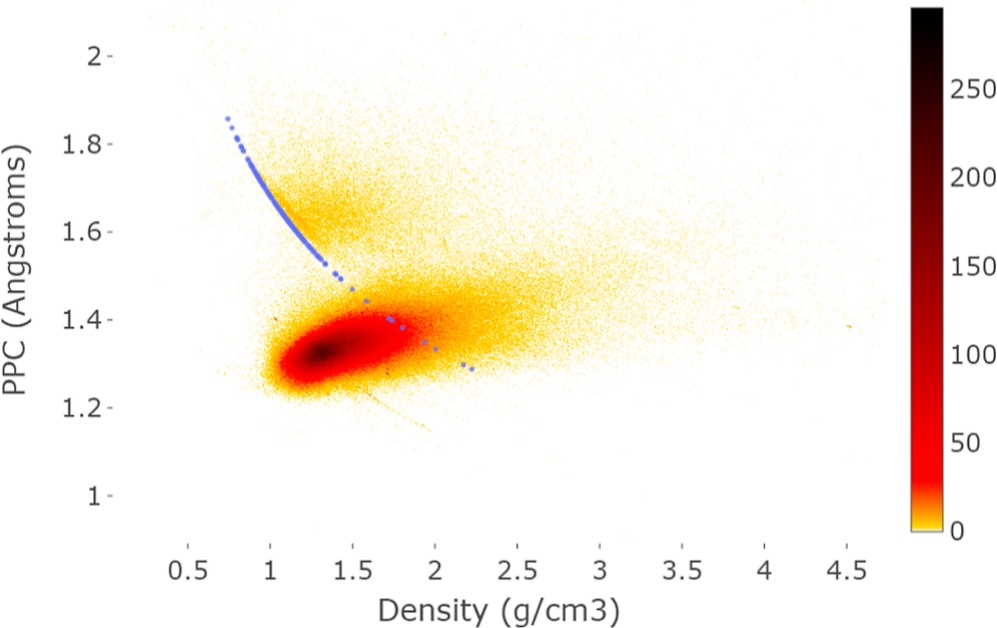
Scatter plot of 208 carbon allotropes over the whole CSD heatmap. The lower dense arc represents compositions H_2_CO, H_5_C_2_NO_2_, and H_6_C_3_NO_3_ with close values of the average atomic mass ATM(*S*). We zoomed on the densest part in the scatter plot in Figure 7.

**Figure 7:**
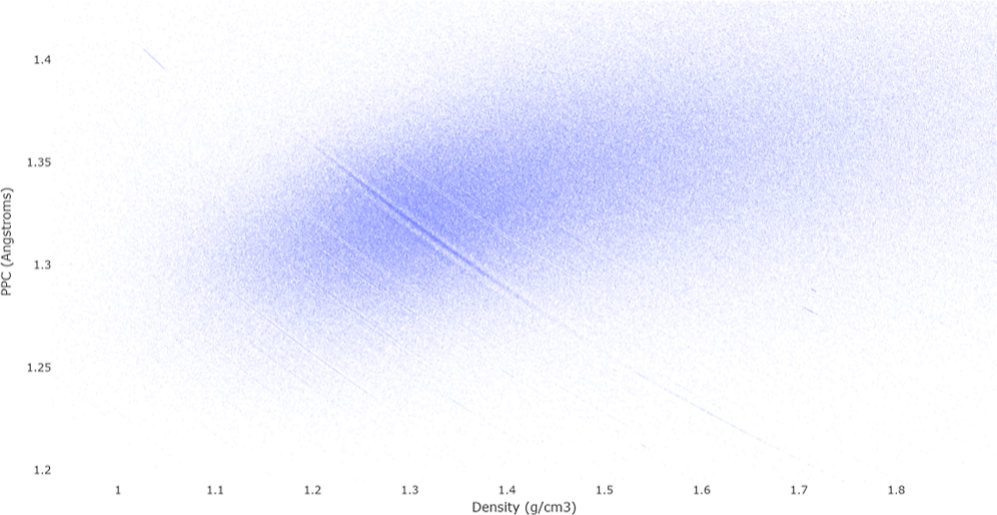
Scatter plot of the densest region in the CSD heatmap from Figure 6 shows how the physical density  depends on the PPC (*S*).

The CRISP is inspired by Richard Feynman’s visual hint in Figures 1–7 of his first lecture (atoms and motion) on physics,^46^ which showed that 7 cubic crystals differ by their only geometric parameter (the smallest interatomic distance) given up to 0.01 Å. Comparing these 7 numbers was the Eureka moment for the last author in May 2021 and motivated to complete all-vs-all comparisons of real periodic crystals in the CSD only by their geometry.

The CRISP does not claim that any periodic set of points can be realized as a real crystal because interatomic distances cannot be arbitrary. Hence, Problem 6 can be strengthened by adding the realizability condition requiring an explicit parametrization of all values *I*(*S*) that can be realized first by a periodic structure *S* and then by a real crystal. This realizability has been achieved for lattices in dimensions two^10^ and three.^26^ In the finite case, any numbers 0 < *a* ≤ *b* ≤ *c* are realizable as distances between *m* = 3 points in  if and only if *c* ≤ *a* + *b*.

However, six interpoint distances between any four points in  satisfy a complicated polynomial equation saying that the tetrahedron on these four points has volume 0 and hence cannot be easily sampled. For example, six interpoint distances 1 satisfy all triangle inequalities and are realizable by an equidistant tetrahedron but not by four points in .

Because the PDD invariants quickly distinguished all real periodic crystals, any such crystal already has a uniquely defined location in the continuous space . So, the CSD can be considered a very big and important discrete subset of . Any newly discovered periodic crystal will appear at a new place of  without disturbing all known ones.

## Continuous Geographic-Style Maps of the CSD

This section presents the first maps of the CSD as a subset in the crystal isometry space  in pairs of invariant coordinates. Big datasets of simulated crystals were often visualized as a structure–energy landscape, which was a scatter plot with two coordinates (density and energy), so the structure was represented by a single invariant. We complement this physical density (g/cm^3^) by the Point Packing Coefficient PPC(*S*) in Definition 7(b).

Figure 6 shows that the physical density and PPC(*S*) differ. A periodic crystal *S* with a unit cell *U* containing atoms whose total mass is mass (*U*) has the physical density
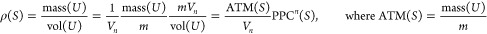
is the average atomic mass (invariant of a chemical composition) of *S*. If we fix a chemical composition of *S* for *n* = 3, so ATM(*S*) and  are fixed, then ρ(*S*) is inversely proportional to PPC^–3^ (*S*), which is confirmed by all carbon allotropes (crystals consisting of pure carbon) whose points [ρ(*S*), PPC(*S*)] lie on a cubic hyperbola in Figure 6.

We zoomed in the central part of all images and excluded outliers beyond the visible ranges, e.g. all crystals with physical densities higher than 4.5 g/cm^3^ are removed in Figure 6.

The scatter plot in Figure 7 further zooms the highest density (black) region from Figure 6.

All maps were produced by our Crystal Geomaps app, which already covers the well-known databases CSD, COD, ICSD, MP, and GNoME, and we can include your data by request. The app is not yet public due to the ongoing IP commercialization discussions with the CCDC. We are also open to collaboration with other partners in industry and academia.

The Supporting Information of this paper contains more maps produced by the Crystal Geomaps app, which allows one to interactively explore the major databases (CSD, COD, ICSD, MP, and GNoME), individual CIFs or user-uploaded data sets of simulated crystals.

Because the average distance AMD_*k*_ to the *k*-th neighbor increases with respect to *k*, the maps with coordinates *x*, *y* from the list AMD_1_ ≤ AMD_2_ ≤ AMD_3_ ≤... are restricted to the half-plane *x* ≤ *y*. To avoid this artificial restriction, we subtracted the limit curve  from AMD_*k*_(*S*) to get the less restrictive invariants ADA_*k*_(*S*) in Definition 7(c).

Figure 10 shows that projections of the CSD to the pairs of invariant coordinates (ρ, PPC) and (ADA_1_, ADA_2_) are very different and hence represent different structural data.

**Figure 8:**
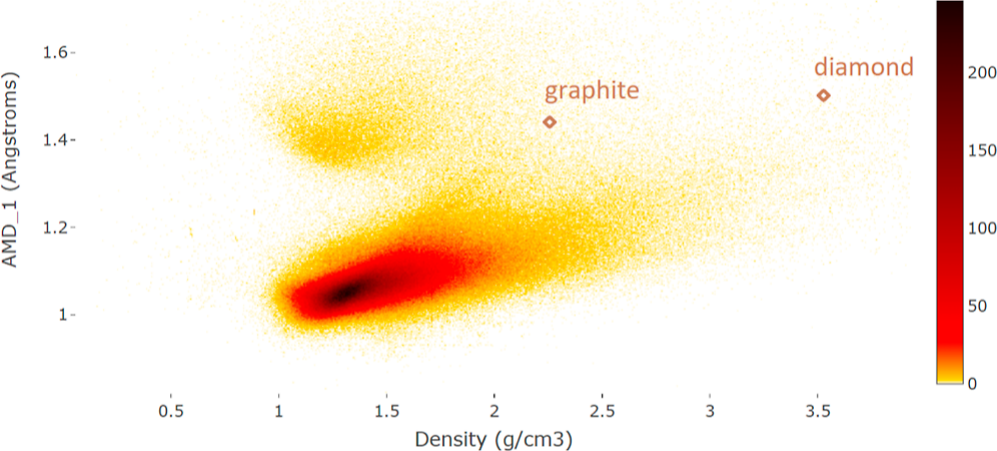
Clusters separated by a gap of AMD_1_ ∈ [1.2, 1.3] are explained by the presence and absence of hydrogens that make AMD_1_ smaller and larger, respectively, see Figure 9.

**Figure 9:**
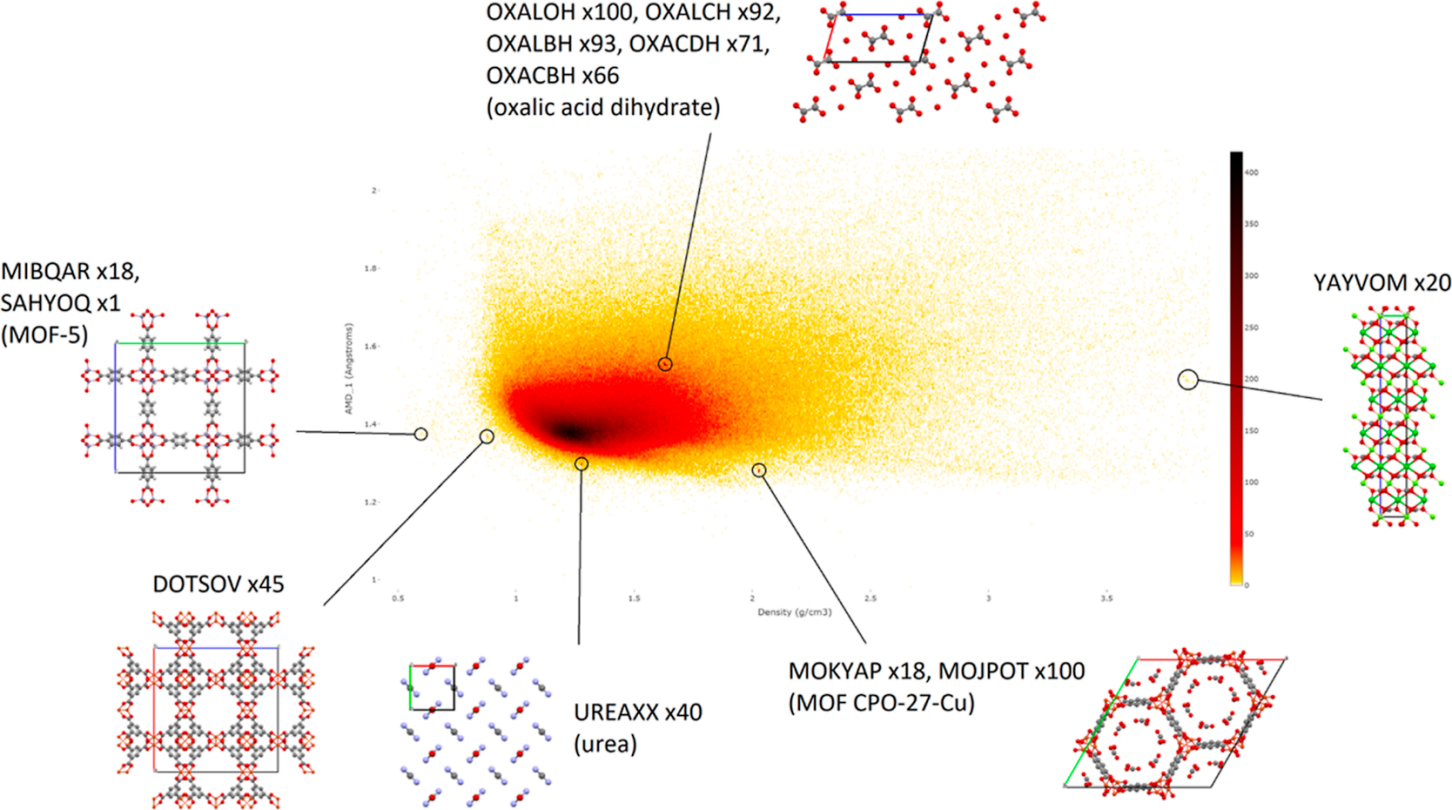
After removing hydrogens, the CSD becomes one large cluster, see Figure 8 in the same coordinates (ρ, AMD_1_). Many isolated dark spots represent groups of many (near-) duplicates.

**Figure 10:**
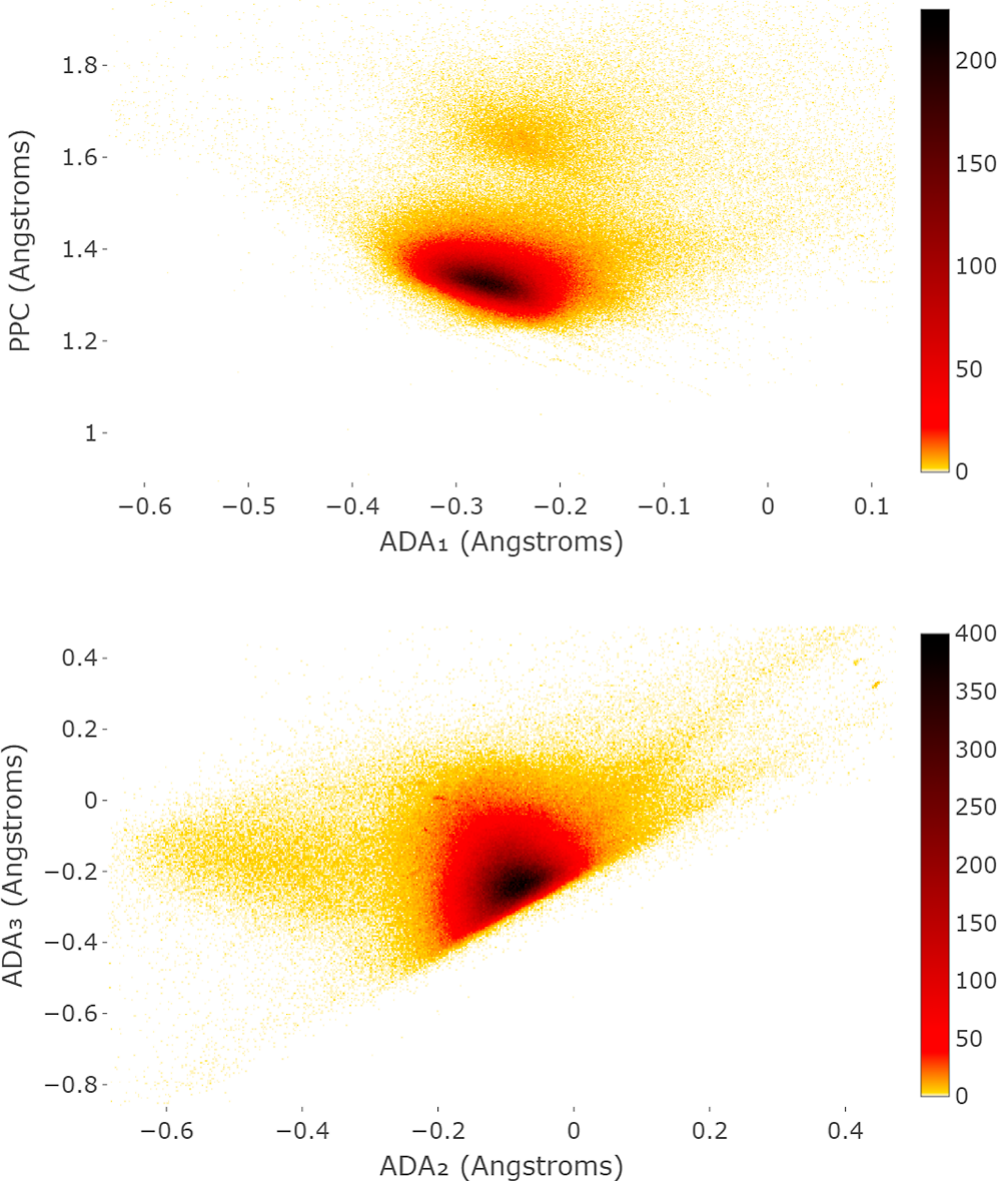
CSD heatmap in the new coordinates PPC, ADA_1_, ADA_2_, and ADA_3_.

Some intermetallic compounds can have close geometries and might appear close neighbors in the space  but we conjecture that all of them can be distinguished if we know the atomic coordinates precisely enough under the same ambient conditions as always.

## Conclusions: Explore A Continuous Universe of Crystals

When Olga Kennard established the CSD nearly 60 years ago, the crystallographic world was much smaller both in terms of people and crystals. Today, crystals are determined by many more methods and produced by artificial tools,^47^ alongside “paper mills”,^48^ claiming new materials without sufficient evidence.^49^ The integrity of major databases^50^ including the CSD^6^ can now be validated by AMD^33^ and PDD invariants.^32^

Figure 3 illustrates how tiny perturbations can disguise any periodic crystal by making any extended cell primitive and pushing any symmetry down to the translation group P1. Because these changes can only slightly affect interatomic distances, then replacing chemical elements with similar ones may not raise any alarm. In November 2023, Google published^11^ the GNoME database of 384,398 CIFs that were claimed to be “stable” materials generated through DFT computations. All-vs-all comparisons will be discussed in another work but anyone right now can check that the four CIFs with IDs 4135ff7bc7, 6370e8cf.86, c6afea2d8e, and e1ea534c2c are identical texts. The GNoME contains 43 such triples, 1089 pairs, and many more thousands of numerical duplicates^12^ that differ only by chemistry, not by geometry.

All images in this previous section are similar to usual geographic maps because they are deterministic projections of the infinite-dimensional crystal isometry space  to pairs of coordinates that are invariant under isometry and rigid motion. All these coordinates have analytic definitions and physically meaningful units such as Angstroms. This interpretability is a key advantage of the new maps in comparison with the past approaches. For example, many algorithms of dimensionality reduction such as t-SNE^51^ and UMAP^52^ are stochastic so that they can produce different outputs by running at different times and on other computers. Even the deterministic algorithms such as regression^53^ and PCA^54^ have data-dependent coordinates and can be discontinuously affected by noise. In 2016, mathematicians proved^55^ that any function  for all *m* > *n* (reducing the dimension from *m* to *n*) is either discontinuous (makes close points distant) or collapses an unbounded region to one point (loses an infinite amount of data) similar to the projection (*x*, *y*) → *x*.

This “no-free-lunch” result implies that a similarity analysis for any high-dimensional data can be justified only by distance metrics in the original high-dimensional space, e.g., by EMD on invariants PDD (*S*; *k*) with *k* up to 100, while low-dimensional projections help visualize data but cannot confirm that any given objects are close.

All crystals whose simplest invariants fall into a single pixel in the maps of Figures 8 and 9, can be visualized with further invariant coordinates ADA_2_(*S*), ADA_3_ (*S*) and so on. This gradual expansion (or zooming in) guarantees that all crystals eventually become distinct because the vector AMD (*S*; 100) distinguished all periodic crystals in the CSD.

Because all maps used only two of many available invariants, we cannot claim that crystals at close positions such as [PPC(*S*), ADA_1_(*S*)] are always similar. However, these invariants quickly filter out dissimilar crystals so that if *S* and *Q* have distant values of ADA_*k*_(*S*), then *S*, *Q* cannot be made identical by a small perturbation of atoms. Due to the Lipschitz continuity, any value δ = EMD (PDD(*S*; *k*), PDD(*Q*; *k*)) > 0 means that we need to shift all atoms by at least by δ/2 on average to fully match the crystals *S*, *Q*.

In conclusion, we motivated studying periodic crystals under much stronger equivalences (rigid motion and isometry) that distinguish many crystals that were previously considered similar or isostructural as in Table 1. In the 20th century, symmetries importantly helped to determine many 3-dimensional structures from their diffraction patterns, especially for highly symmetric crystals. In 2024, the ultrafast PDD invariants allowed us to move beyond the 230 space groups in  toward the continuous universe containing all known periodic crystals (already visible “stars” on our maps) and also all not yet synthesized ones.

Visualizing important properties such as energy as mountainous landscapes on these maps will help distinguish between shallow local minima and more stable materials in deeper “wells” surrounded by energy barriers. Now, all crystal structures at least in the CSD are fully discriminated by a series of Lipschitz continuous PDD invariants. This solution of the discriminative problem justifies a generative approach to explore new PDD matrices, which are always guaranteed to be realizable by at most one real crystal whose properties are unique. Further work will continuously quantify the novelty of any newly discovered crystal by a distance metric to its closest structural neighbor across all experimental databases.

The CRISP and underlying invariants were presented at the IUCr congresses 2021 and 2023, European Crystallographic Meeting 2022, British Crystallographic Association meetings 2022–2024, MACSMIN 2021–2023 (Mathematics and Computer Science for Materials Innovation), SIAM Mathematical Aspects of Materials Science conferences 2021 and 2024, and many MIF++ seminars at the Materials Innovation Factory in Liverpool, UK.

## Supporting Information Available

Functionality of the crystal geomaps app and continuous maps of some CSD subsets of crystals with specific chemical compositions (PDF)
