# Supplementary material for: Continuous Invariant-Based Maps of the Cambridge Structural Database
Source: Cryst Growth Des. 2024 Jun 20;24(13):5627–36. doi: 10.1021/acs.cgd.4c00410 (PMC11228915; doi:10.1021/acs.cgd.4c00410)
Supplement: Supplementary file 1 — cg4c00410_si_001.pdf [file cg4c00410_si_001.pdf]

Supporting Information for Publication: Continuous invariant-based maps of the Cambridge Structural Database

Daniel E Widdowson and Vitaliy A Kurlin*

Materials Innovation Factory and Department of Computer Science, University of Liverpool, Liverpool L69 3BX, United Kingdom

E-mail: vitaliy.kurlin@liverpool.ac.uk

Phone: +44 (151) 7958861

Fig. S1 shows two parts of the menu for the Crystal Geomap app. Hovering the mouse over any pixel in a heatmap shows the coordinates x, y and the number of crystals at this position (x, y). Hovering the mouse over any dot (x, y) in a scatter plot shows the database reference, composition, and space group number of the crystal at (x, y).

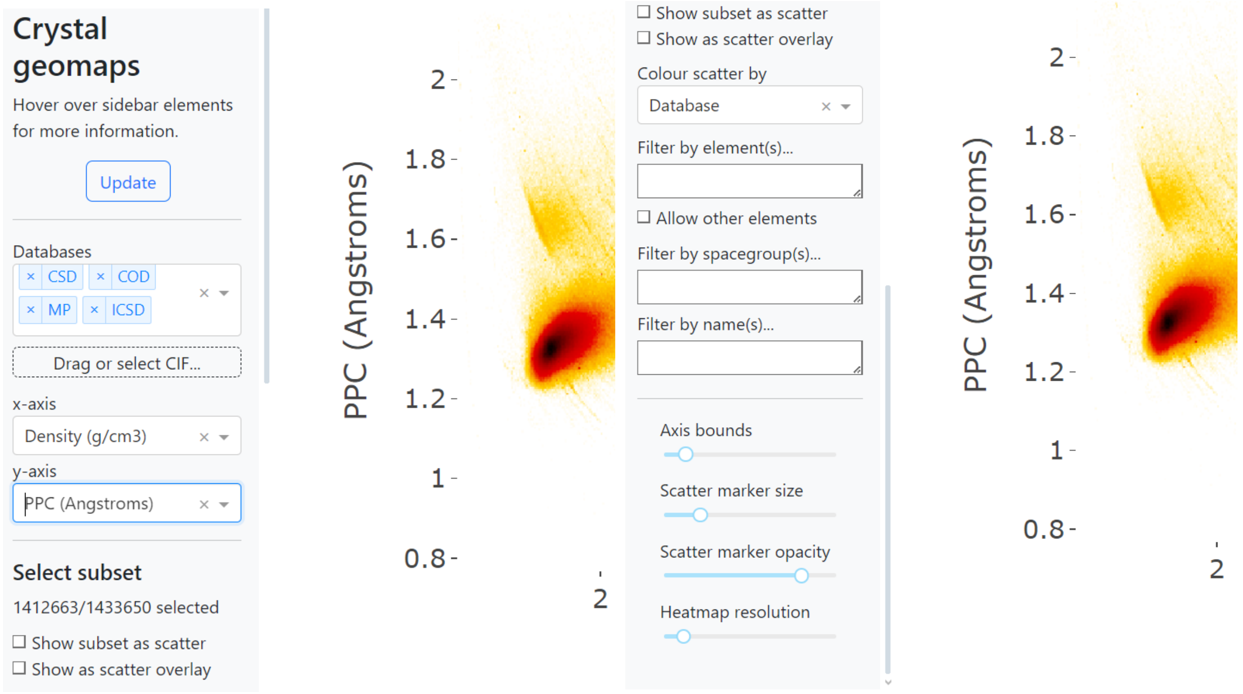

Fig. S1: The images on the left and right show two parts of a user menu with choices for coordinates (density, PPC, ADA$_k$, NDA$_k$) and display options. A scatter plot represents any crystal from a selected subset by an individual dot and can also be plotted over a density heatmap of chosen databases. A subset can be selected by chemical elements, space groups, e.g. 195-230 for the cubic crystal system, or database reference names.

Fig. S1 implies that the total number of ideal periodic crystals (with no disorder) in the four major databases is more than 1,433,000, though many of them are (near-)duplicates often deposited from the same publication. These overlaps between different databases will be analysed in another work. The displayed number of crystals is slightly smaller because some outliers are outside the visible ranges that can be adjusted by the slider "Axis bounds". Other sliders control sizes of dots and pixels. In any scatter plot, all crystal dots can be coloured by a database name, 7 crystal systems, 230 space groups and invariant values.

Further Figures S3, S4, S5, S6 show some smaller interesting subsets of the CSD.

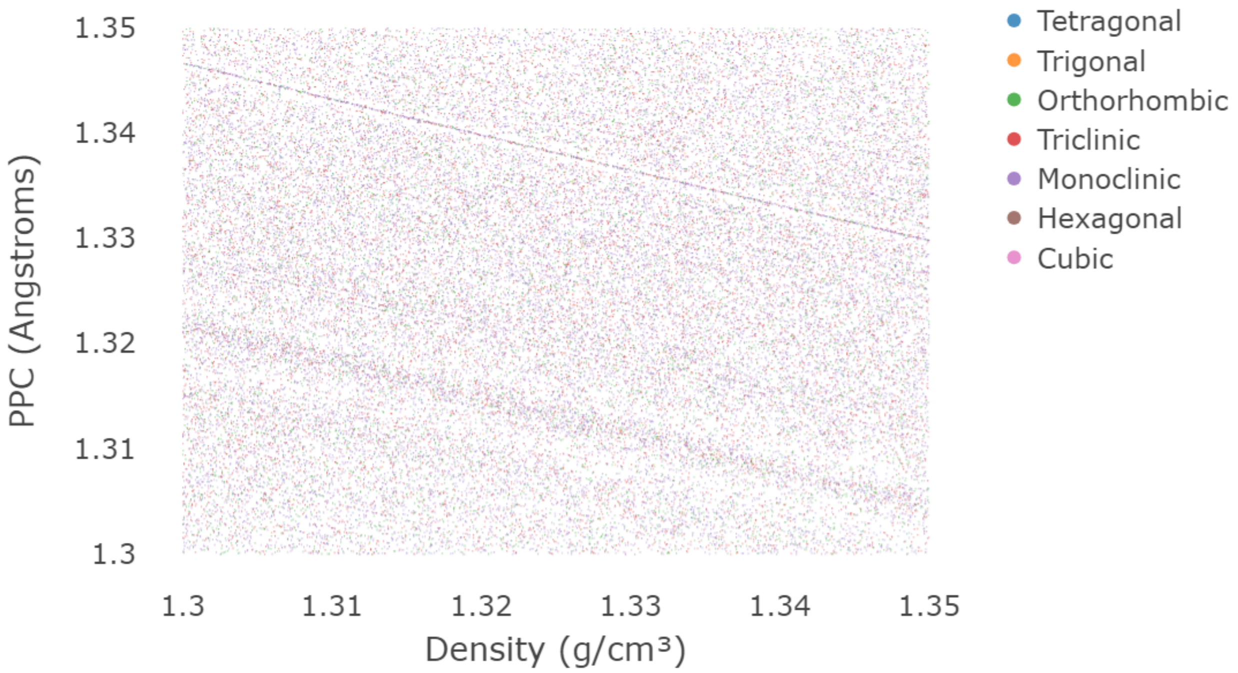

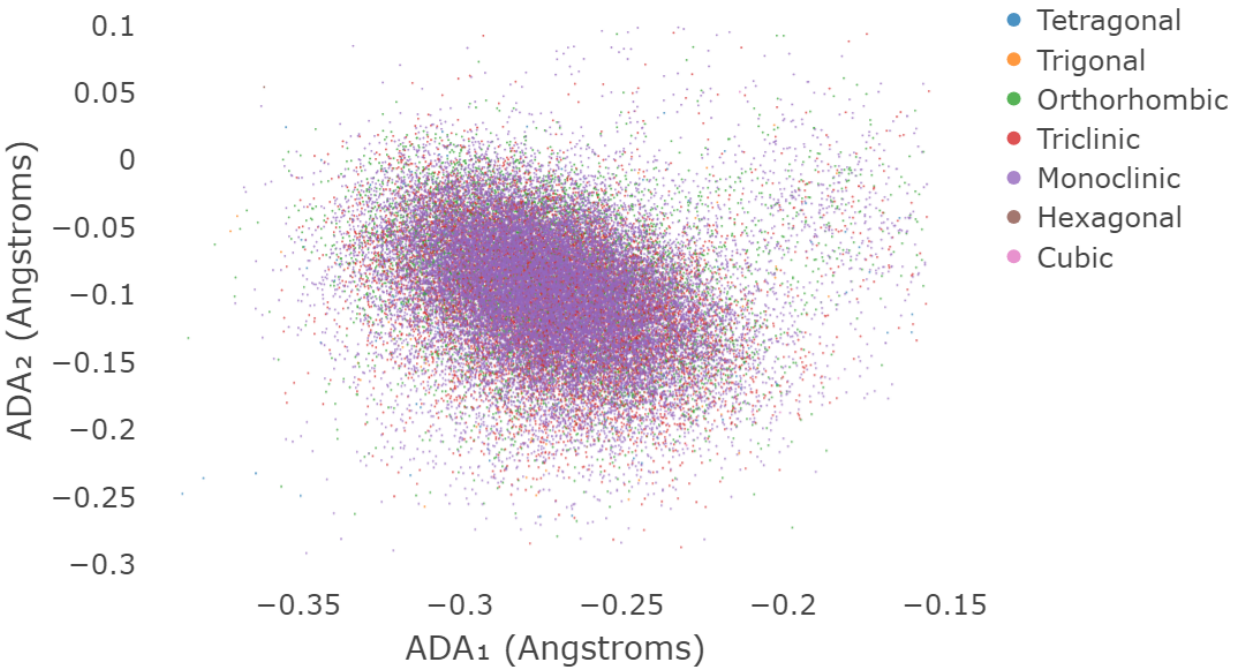

Fig. S2: **Top**: the densest rectangular region of 44102 crystals from the CSD in the coordinates (ρ, PPC). **Bottom**: the same subset is differently projected to $(\mathrm{ADA}_1, \mathrm{ADA}_2)$.

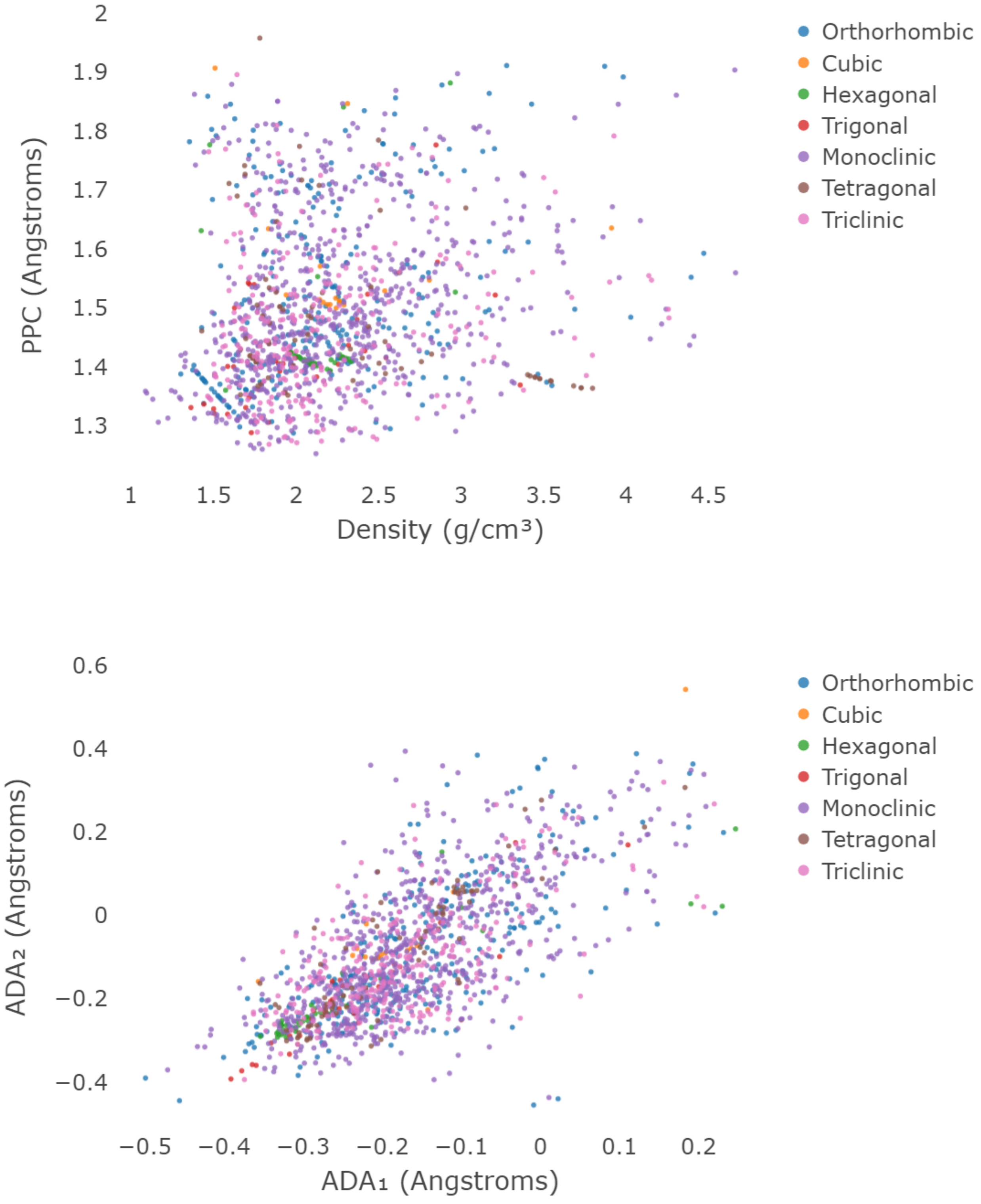

Fig. S3: 1453 crystals containing carbon and sulfur, all coloured by their crystal systems.

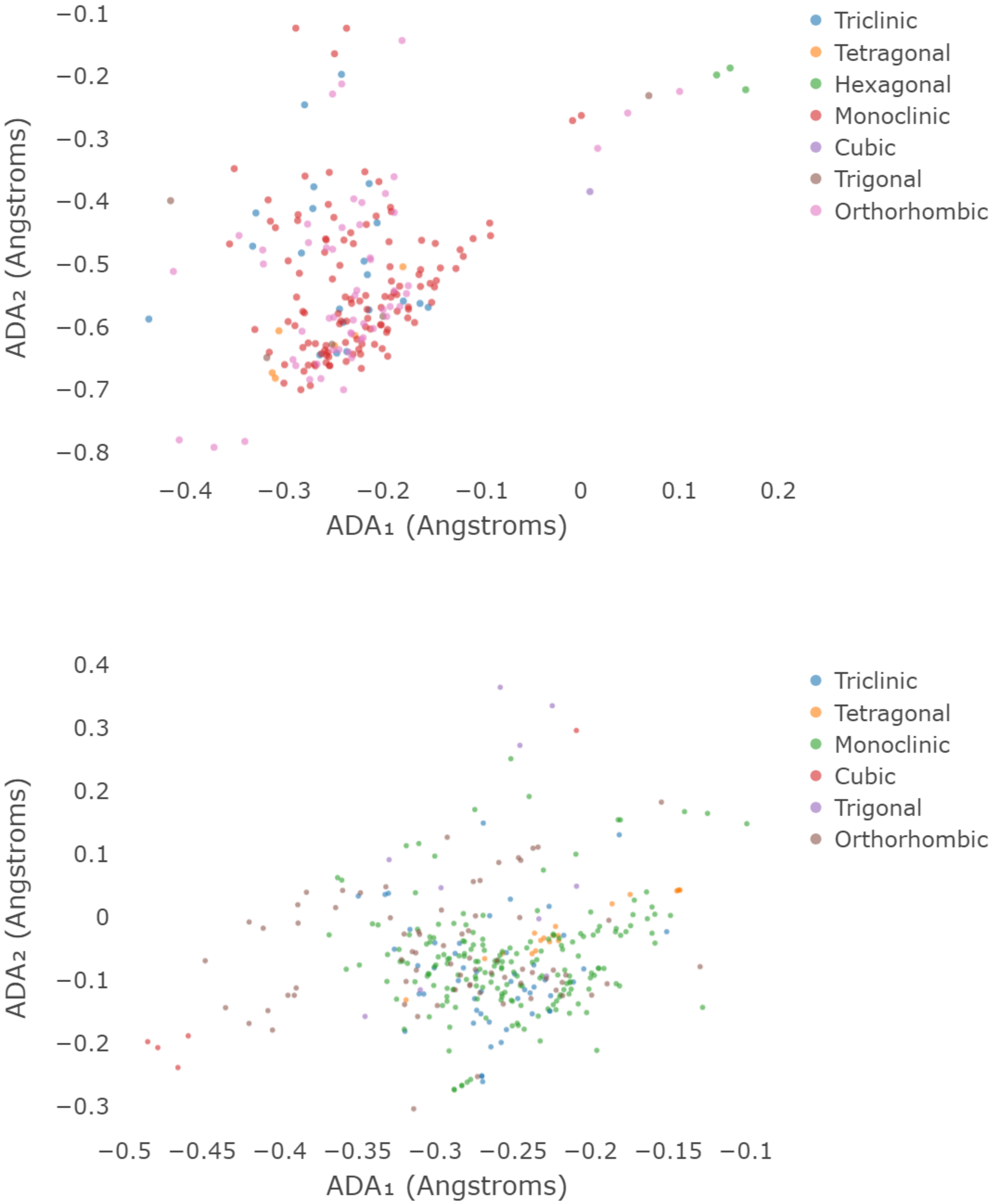

Fig. S4: **Top**: the scatter plot of 208 carbon allotropes in the CSD. **Bottom**: the scatter plot of 345 carbohydrates in the CSD, all coloured by their crystal systems.

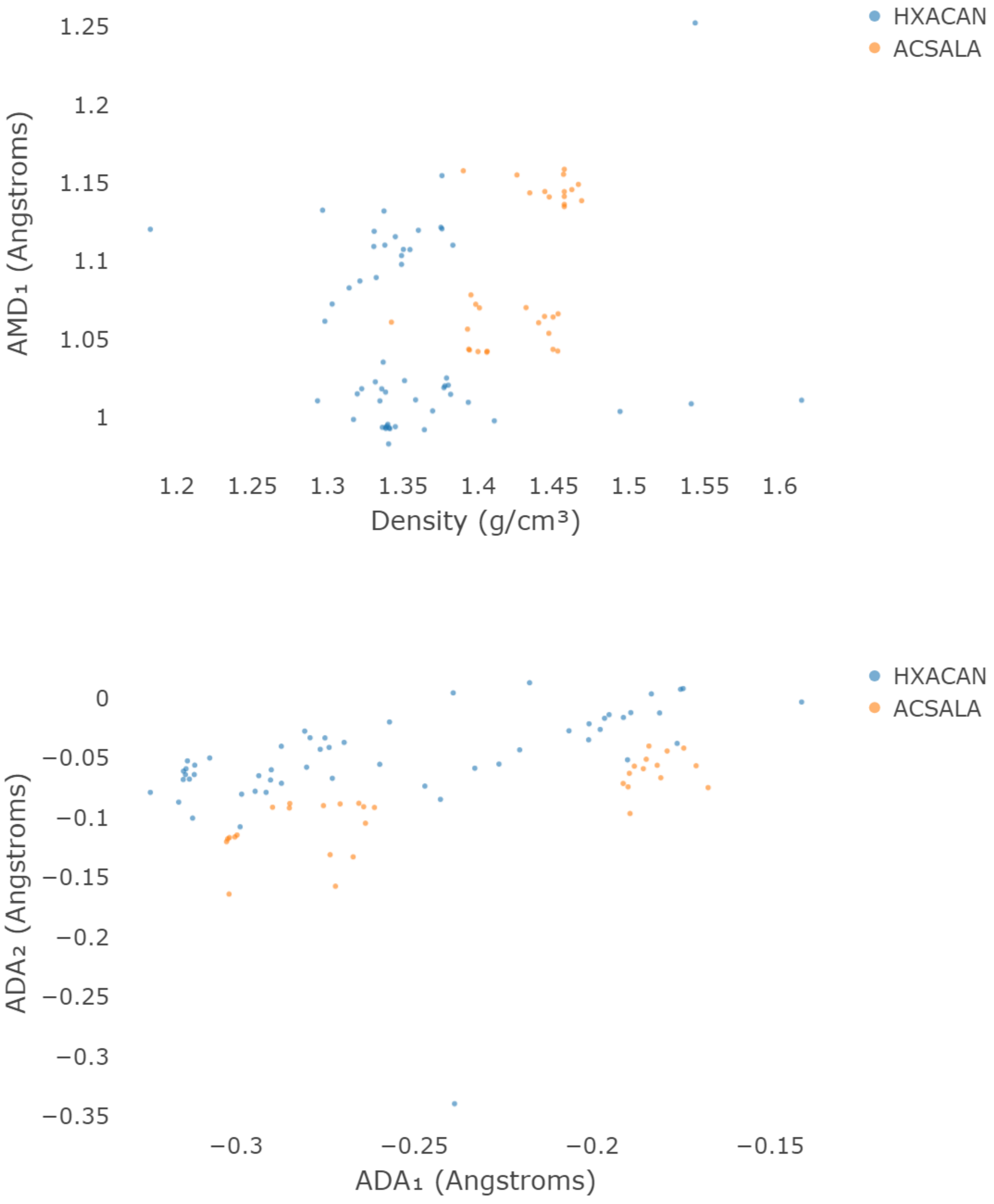

Fig. S5: 86 crystals of ACSALA (aspirin) and HXACAN (paracetamol) in the CSD.

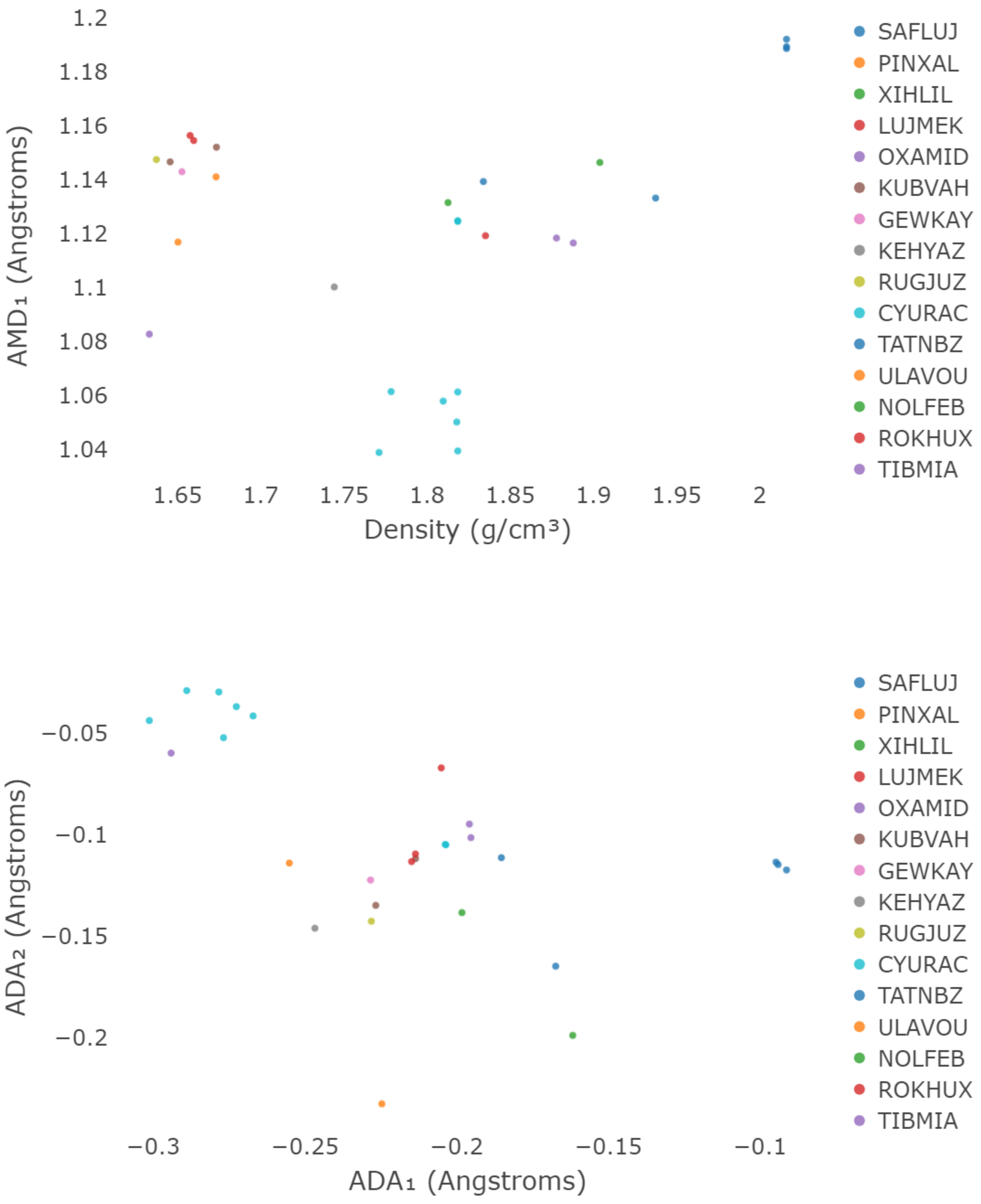

Fig. S6: 28 crystals containing only C, O, H, N, all coloured by CSD refcode families.
